# Genome-wide association study for systemic lupus erythematosus in an egyptian population

**DOI:** 10.3389/fgene.2022.948505

**Published:** 2022-10-17

**Authors:** Ashraf A. Elghzaly, Celi Sun, Loren L. Looger, Misa Hirose, Mohamed Salama, Noha M. Khalil, Mervat Essam Behiry, Mohamed Tharwat Hegazy, Mohamed Ahmed Hussein, Mohamad Nabil Salem, Ehab Eltoraby, Ziyad Tawhid, Mona Alwasefy, Walaa Allam, Iman El-Shiekh, Menattallah Elserafy, Anwar Abdelnaser, Sara Hashish, Nourhan Shebl, Abeer Abdelmonem Shahba, Amira Elgirby, Amina Hassab, Khalida Refay, Hanan Mohamed El-Touchy, Ali Youssef, Fatma Shabacy, Abdelkader Ahmed Hashim, Asmaa Abdelzaher, Emad Alshebini, Dalia Fayez, Samah A. El-Bakry, Mona H. Elzohri, Eman Nagiub Abdelsalam, Sherif F. El-Khamisy, Saleh Ibrahim, Gaafar Ragab, Swapan K. Nath

**Affiliations:** ^1^ Department of Clinical Pathology, Faculty of Medicine, Mansoura University, El-Mansoura, Egypt; ^2^ Arthritis and Clinical Immunology Research Program, Oklahoma Medical Research Foundation, Oklahoma City, OK, United States; ^3^ Department of Neurosciences, Howard Hughes Medical Institute, University of California, San Diego, San Diego, CA, United States; ^4^ Division of Genetics, Lübeck Institute of Experimental Dermatology, University of Lübeck, Lübeck, Germany; ^5^ Institute of Global Health and Human Ecology, The American University in Cairo, New Cairo, Egypt; ^6^ Rheumatology and Clinical Immunology Unit, Department of Internal Medicine, Faculty of Medicine, Cairo University, Cairo, Egypt; ^7^ Department of Internal Medicine, Faculty of Medicine, Beni-Suef University, Beni Suef, Egypt; ^8^ Department of Internal Medicine, Faculty of Medicine, Mansoura University, El-Mansoura, Egypt; ^9^ Center for Genomics, Helmy Institute for Medical Sciences, Zewail City of Science and Technology, Giza, Egypt; ^10^ Department of Internal Medicine, Faculty of Medicine, Tanta University, Tanta, Egypt; ^11^ Department of Internal Medicine, Faculty of Medicine, Alexandria University, Bab Sharqi, Egypt; ^12^ Department of Clinical Pathology, Faculty of Medicine, Alexandria University, Bab Sharqi, Egypt; ^13^ Department of Internal Medicine, Faculty of Medicine, Al-Azhar University, Cairo, Egypt; ^14^ Department of Rheumatology and Immunology, Faculty of Medicine, Benha University Hospital, Benha, Egypt; ^15^ Department of Internal Medicine, Faculty of Medicine, South Valley University, Qena, Egypt; ^16^ Department of Clinical Pathology, Faculty of Medicine, South Valley University, Qena, Egypt; ^17^ Department of Internal Medicine, Faculty of Medicine, Menoufia University, Al Minufiyah, Egypt; ^18^ Rheumatology and Clinical Immunology Unit, Department of Internal Medicine, Faculty of Medicine, Ain Shams University, Cairo, Egypt; ^19^ Department of Internal Medicine, Faculty of Medicine, Assiut University, Asyut, Egypt; ^20^ Department of Clinical Pathology, Faculty of Medicine, Assiut University, Asyut, Egypt; ^21^ The Healthy Lifespan Institute, University of Sheffield, Sheffield, United Kingdom; ^22^ The Institute of Cancer Therapeutics, University of Bradford, Bradford, United Kingdom

**Keywords:** GWAS, lupus, admixture, Egypt, imputation

## Abstract

Systemic lupus erythematosus (SLE) susceptibility has a strong genetic component. Genome-wide association studies (GWAS) across trans-ancestral populations show both common and distinct genetic variants of susceptibility across European and Asian ancestries, while many other ethnic populations remain underexplored. We conducted the first SLE GWAS on Egyptians–an admixed North African/Middle Eastern population–using 537 patients and 883 controls. To identify novel susceptibility loci and replicate previously known loci, we performed imputation-based association analysis with 6,382,276 SNPs while accounting for individual admixture. We validated the association analysis using adaptive permutation tests (*n* = 10^9^). We identified a novel genome-wide significant locus near *IRS1/miR-5702* (P_corrected_ = 1.98 × 10^−8^) and eight novel suggestive loci (P_corrected_ < 1.0 × 10^−5^). We also replicated (P_perm_ < 0.01) 97 previously known loci with at least one associated nearby SNP, with *ITGAM, DEF6-PPARD* and *IRF5* the top three replicated loci. SNPs correlated (*r* ^2^ > 0.8) with lead SNPs from four suggestive loci (*ARMC9, DIAPH3*, *IFLDT1,* and *ENTPD3*) were associated with differential gene expression (3.5 × 10^−95^ < *p* < 1.0 × 10^−2^) across diverse tissues. These loci are involved in cellular proliferation and invasion—pathways prominent in lupus and nephritis. Our study highlights the utility of GWAS in an admixed Egyptian population for delineating new genetic associations and for understanding SLE pathogenesis.

## 1 Introduction

Systemic lupus erythematosus (SLE) is a chronic, complex, multi-system autoimmune disease with substantial mortality and morbidity. Prevalence, severity, and sub-clinical manifestations vary significantly across ethnically diverse populations—with an outsized burden on individuals with African, Hispanic, and Asian backgrounds compared to Caucasians (Carter et al., 2016; Lewis and Jawad, 2017; Pisetsky et al., 2017). Additionally, SLE shows remarkable gender bias, affecting ∼9–10 times as many women as men.

In addition to epigenetic and environmental contributions, SLE has a very strong genetic component. This is evidenced by high heritability (∼66%), familial clustering (sibling recurrence risk ratio ∼30), twin studies (∼10 times higher concordance rate in monozygotic *versus* dizygotic twins) (Deapen et al., 1992; Block, 2006; Mak and Tay, 2014; Kuo et al., 2015), and by a growing number of susceptibility loci identified by genome-wide association studies (GWAS) and high-density candidate gene studies [e.g., ImmunoChip (Cortes and Brown, 2011)]. Despite the identification of ∼180 SLE susceptibility loci (Bentham et al., 2015; Sun et al., 2016; Langefeld et al., 2017; Molineros et al., 2017; Julia et al., 2018; Wang et al., 2018; Tangtanatakul et al., 2020; Ha et al., 2022), these account for only ∼30% of genetic heritability (Yin et al., 2020), indicating that many genes and pathways remain incompletely mapped or even undiscovered.

The statistical power and locus resolution of GWAS and candidate-gene studies can be increased in several ways: larger sample size, deeper genotyping of existing samples, improved imputation and statistical fine-mapping, and most importantly, introducing samples with different allele and haplotype usage than existing samples. Critically, most lupus studies—and most genetic studies in general—are built mainly upon individuals with European (and to a lesser extent Asian) ancestries (Ha et al., 2022). However, many alleles occur at low frequencies in these ethnicities, preventing statistically meaningful measurement of their effects. The use of underrepresented ethnicities with different allelic usage and recombination hotspots is the single most effective way to increase association power (Wojcik et al., 2019). In addition to providing sufficient power to resolve additional ancestry-independent signals, such studies also illuminate ancestry-specific signals (Goulielmos et al., 2018)—wherein a locus is risk only in a specific ethno-genetic background. Such studies are particularly important in autoimmune diseases like lupus, which afflict non-white ethnicities at much higher rates than whites.

Lack of diversity in the available human genomes deposited in public databases limits our understanding of the genetic underpinnings of complex traits, hinders precision medicine, and contributes to health disparities (Popejoy and Fullerton, 2016; Ben-Eghan et al., 2020). Africans and Middle Easterners are among the populations the most underrepresented in genetic association studies, despite comprising over 1.5 billion people and coming from the birthplace of humanity. Moreover, Africa is home to more genetic diversity than the rest of the world combined (Tishkoff et al., 2009) and as such constitutes a woefully underutilized pool for genetic discoveries. Meanwhile, the Middle East and North Africa were both the cradle of civilization and the largest crossroads of migration in the ancient world, bringing together Africa, Europe, and Asia as empires rose and fell. A recent population admixture study (Schuenemann et al., 2017) showed that Ancient Egyptians were more closely related to Middle Easterners and Europeans than to Africans, with African ancestry increasing after the fall of the Roman Empire. In SLE, striking racial/ethnic differences exist in incidence, disease course and clinical manifestations; genetic studies will be helpful to delineate these disparities. However, very few genetic association studies on Egyptians have been performed thus far—and most feature only a few SNPs from a handful of candidate genes (Elghzaly et al., 2015). Therefore, a large-scale genome-wide wide association study is required to understand the SLE genetic landscape in Egyptians—here, we perform the first such study. The major findings of our study are: 1) nine newly uncovered SLE susceptibility loci with one genome-wide significant, 2) replication of several previously known SLE loci, and 3) remarkable similarities in minor allele frequencies at risk loci between Egyptians and Europeans.

## 2 Materials and methods

### 2.1 Systemic lupus erythematosus patients and controls

**FIGURE 1:**
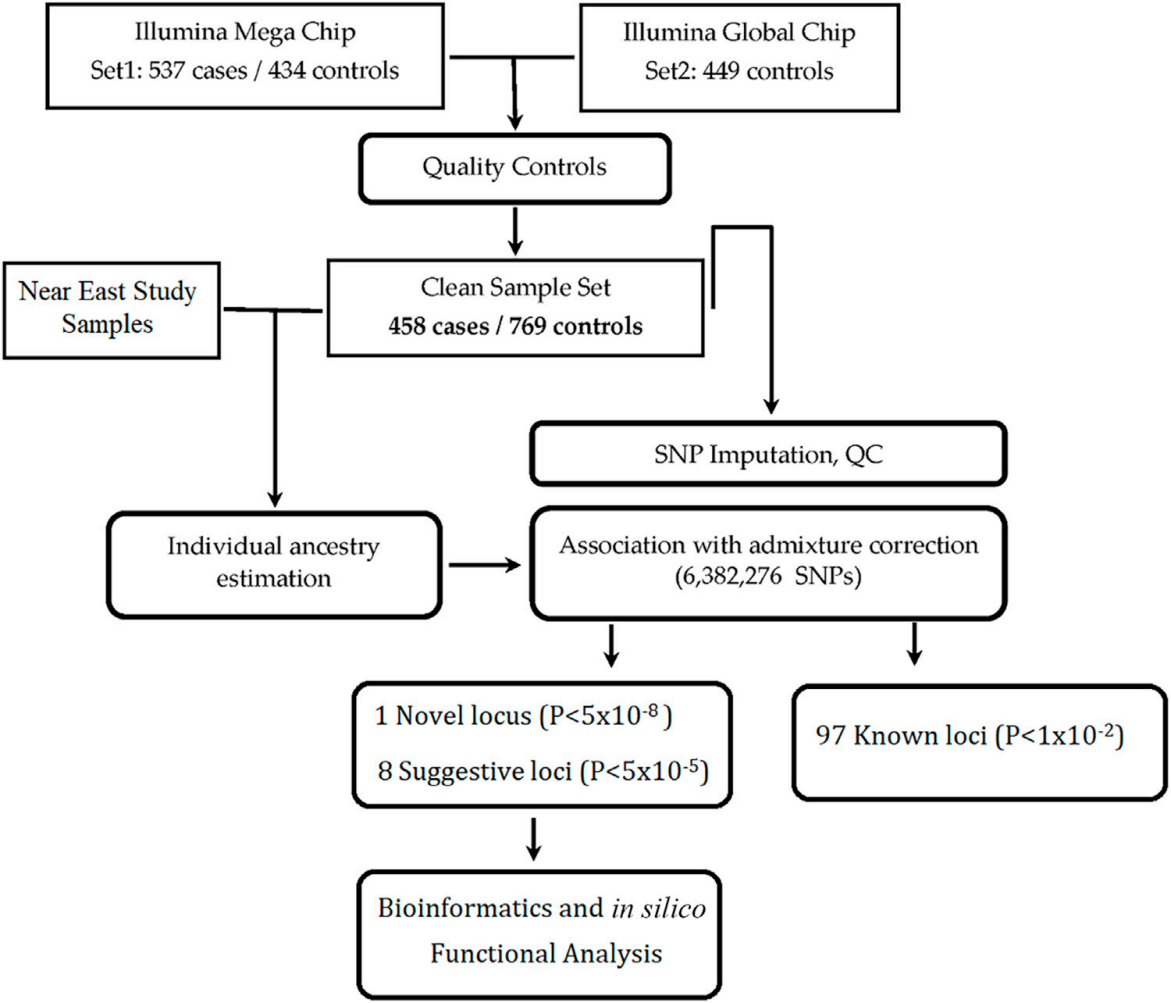
Overview and study design.

The overview and design of the study is shown in Figure 1. Patients were informed of the nature of the study, and only those who gave their consent were included in the study. SLE was diagnosed according to the 1997 update of the Revised American College of Rheumatology (ACR) classification criteria for SLE (Hochberg, 1997) or the “Systemic Lupus International Collaborating Clinic” (SLICC) Criteria for Classification of Systemic Lupus Erythematosus (Petri et al., 2012). Our rheumatologists used both diagnostic criteria, because we had patients of long disease history with established diagnosis based on earlier criteria that preceded the SLICC standard. Patients who were identified as SLE positive after the introduction of the SLICC criteria were diagnosed accordingly and the two subsets, being included in each center’s registries, were recruited for the study as definite cases of SLE. Note that we lack ACR/SLICC information and some demographic data for 26 SLE patients who have left physicians’ care and are no longer available for verification. Patients’ recruitment, clinical examinations, diagnostic laboratory investigations, and data collection were all performed in their corresponding medical centers by expert rheumatology teams and in specialized university laboratories. Data was collected in an *ad hoc* unified Excel spreadsheet and reviewed by senior rheumatology experts. Exclusion criteria included the presence of overlap features or the presence of other autoimmune diseases except for Sjögren’s syndrome. The control group was selected from the same geographical location as the corresponding center. All participants provided informed consent, and the study was approved by the Institutional Review Boards from Oklahoma Medical Research Foundation (OMRF), Oklahoma City, United States (IRB approval number 14–16), and Mansoura University Ethics committee/IRB (MFM-IRB code number R/16.04.81). The recruiting hospitals/universities included Cairo University, Mansoura University, Tanta University, Alazhar University–females only, Beni-Suef University, Benha University, Menofia University, Alexandria University, Ain Shams University, South Valley University, and Assuit University.

Initially, 537 Egyptian SLE patients and 434 controls were recruited for the GWAS study, and DNA samples were extracted from 5 ml whole blood samples collected in EDTA-containing tubes and stored at −20°C. DNA extraction for all samples was done in Germany, according to manufacturer’s instructions using the Qiamp DNA Blood Mini Kit (QIAGEN, Hilden, Germany). To increase statistical power, 449 additional unaffected Egyptian controls [already genotyped in Germany for another GWAS study (Bejaoui et al., 2019)] were added to the study. These controls were recruited from the blood banks of Cairo and Mansoura Universities and approved by the IRBs of both Universities (as mentioned above).

### 2.2 Demographics

Gender and age distributions for SLE cases and controls (after quality control, described below) are shown in Supplementary Table S1. Overall, females constituted 90% and 64% of cases and controls, respectively. Average ages of cases and controls are comparable. The distribution of major ACR criteria within SLE patients are shown in Supplementary Table S1. Almost all (99%) SLE patients were positive for anti-nuclear antibodies (ANA).

### 2.3 Genotyping and quality control

Initial samples (537 SLE patients and 434 controls) were genotyped on the Illumina Multi-Ethnic Genotyping Array (MEGA) at the genotyping core facility of OMRF, Oklahoma City, United States. The 449 additional out-of-study controls were already genotyped on the Illumina Infinium Global Screening Array. The out-of-study controls have no genotyping data on the X-chromosome; thus we decided to focus only on autosomal loci. We hope to obtain this missing genotyping data for use in future studies on X-linked traits. We performed strict quality control (QC) on genotyped SNPs in each dataset separately (cases and two sets of controls) as follows: (a) we excluded SNPs with bad clusters due to poor genotyping calls, (b) we removed SNPs with missing genotype rate ≥ 0.05, and (c) we filtered out SNPs out of Hardy-Weinberg equilibrium (*p* < 0.0001) and/or minor allele frequency (MAF) < 0.5%. After QC, 295,981 autosomal (chromosomes 1–22) SNPs that overlapped both genotyping arrays remained for further analysis. Of the 537 cases and 883 controls, the GCTA algorithm (Yang et al., 2011) selected 500 cases and 815 controls as being unrelated from one another; the others were excluded. As a final QC step, 42 cases and 46 controls were removed from the cohort due to being outliers from principal components analysis—leaving 458 cases and 769 controls for this study. The inflation factor λ was calculated from the case-control association test on the final, clean cohort (458 cases and 769 controls).

### 2.4 Statistical power for detecting association

To estimate the ability of our GWAS cohort to support statistically significant associations, we performed a statistical power calculation. The Genetic Association Study Power Calculator server (Johnson and Abecasis, 2017), derived from the CaTS power calculator for two-stage association studies (Skol et al., 2006), was used. We used our final cohort (458 cases, 769 controls) to assess statistical power, while setting the parameter prevalence for SLE to 0.3% (Wang Y. F. et al., 2021).

### 2.5 Imputation for GWAS samples

We used the Michigan Imputation Server (Das et al., 2016) for imputation on the 22 autosomes. 1,000 Genomes Project data (Phase 3 Integrated Release Version 5 Haplotypes) was used as the reference panel. After imputation, we performed strict QC on post-imputed SNPs as described above (Hardy-Weinberg equilibrium *p* > 0.0001 in controls and/or MAF ≥ 0.5%). About 6.36 million post-imputed SNPs with high imputation quality (Rsq > 0.7 for MAF ≥ 3%) were used for downstream analysis.

### 2.6 Admixture-corrected association analysis with imputed data

To quantify individual ancestry proportion in our Egyptian cohort, we complemented our dataset with published samples from the ancient Near East (Lazaridis et al., 2016) (the historical Fertile Crescent and Levant regions, corresponding to modern-day Egypt, Turkey, Iran, and surrounding nations.) The Near East samples include 294 ancient and 2,068 modern individuals spanning a range of nationalities and ethnicities. In both the Egyptian dataset and Near East public dataset, we excluded SNPs with A/T or C/G alleles and identified ∼33,000 common unrelated SNPs (MAF > 5% and linkage disequilibrium, LD *r* ^2^ < 0.2) for analysis. Principal components analysis was performed with GCTA (Yang et al., 2011), and genetic ancestry composition was estimated with ADMIXTURE (Alexander et al., 2009).

We performed imputation-based association analyses using mach2dat (Li et al., 2009) before and after admixture correction. (A/T and C/G alleles were recovered during imputation.) ADMIXTURE uses a maximum-likelihood approach to determine admixture proportions of individuals by assuming there are *K* hypothetical underlying populations among them. We selected *K* = 5 as the best model for the number of ancestral populations and took the first four ancestry proportions as covariates for admixture correction. The inflation factor (λ) was estimated using all admixture-corrected association results from QC-passed SNPs.

We performed multiple-testing correction using permutation (Gao, 2011)—considered the gold standard in GWAS studies. Note that we specifically avoided Bonferroni correction as this assumes independence, which is failed when any SNPs are in linkage disequilibrium—thus producing unacceptably conservative associations (Johnson et al., 2010). Besides, Bonferroni correction suffers several shortcomings, most notably low statistical power for rejecting the null hypothesis (Cohen, 1990, 1994; Perneger, 1998; Nakagawa, 2004) and also performing large amounts of unnecessary testing (Fadista et al., 2016). Critically, permutation analysis controls for low sample-size effects, as all permutations have identical *n* as the proposal—comparisons are between permutations with identical statistical power *a priori* to produce an association. Thus, we do not fear spurious associations despite our relatively small sample sizes.

### 2.7 Replication of previously known variants and loci

To replicate previously reported SLE susceptibility loci, we followed a recent study (Ha et al., 2022) that collated 179 statistically-independent non-human leukocyte antigen (HLA) loci as underlying SLE susceptibility. At each risk locus, we defined a boundary as ± 150 kb around the nominated SNP and collated the full set of SNPs in these intervals. Then, we compared this (enlarged) SNP set with the admixture-corrected association results. Using an ancestry-corrected permutation-based analysis, we set the threshold for replication at *P*_perm_ < 0.01—stricter than the more commonly used 0.05 threshold, to better exclude false positive associations.

### 2.8 SNP functional annotation

Functional interpretation of the associated SNP under any GWAS peak is of critical importance in follow-up analysis. Like most other GWAS studies, many of our identified SNPs concentrate in non-coding DNA, e.g., transcriptional regulatory regions (promoters, enhancers) and non-coding RNA. Therefore, analyzing tissue-specific effects of expression quantitative trait loci (eQTLs) is a promising approach. We used Qtlizer (Munz et al., 2020) for the top SNPs in the novel loci to obtain their eQTLs from several public databases, including GTEx v7 (Consortium, 2013), GEUVADIS (Lappalainen et al., 2013), GRASP (Leslie et al., 2014), Haploreg (Ward and Kellis, 2012), SCAN (Gamazon et al., 2009), seeQTL (Xia et al., 2011), Blood eQTL Browser (Westra et al., 2013), pGWAS (Suhre et al., 2017), ExSNP (Yu et al., 2016), and BRAINEAC (Ramasamy et al., 2014). Qtlizer allowed exploration of QTL data for a given list of variants (indels and SNPs) in a fast and efficient manner by integrating many QTL datasets.

### 2.9 Annotation of long non-coding RNA targets and microRNAs

Target sites of lncRNAs were predicted using the LncRRIsearch server (http://rtools.cbrc.jp/LncRRIsearch) (Fukunaga et al., 2019), built upon RIblast (Fukunaga and Hamada, 2017), which computationally identifies lncRNA-mRNA interactions through a seed-and-extension approach. For miRNAs, we identified similar sequences in the human genome (GRCh37/hg19) with BLAT on the UCSC Genome Browser (GRCh37/hg19).

## 3 Results

### 3.1 Study population and statistical power

After rigorous QC, 458 cases and 769 controls were identified by GCTA (Yang et al., 2011) as unrelated samples and were used for association (Figure 1, Section 2). Due to the small sample size, our study had moderate power (68%) for detection of significant (*p* < 1 × 10^−5^) variants with odds ratio (OR) < 1.5 (Supplementary Figure S1). However, for common (minor allele frequency, MAF ≥ 10%) SNPs with large effect (OR ≥ 1.6), our study had high (up to 90%) power. The inflation factor (λ) between cases and controls was 1.06; the QQ plot is shown in Supplementary Figure S2.

### 3.2 Genome-wide association of SLE

**FIGURE 2:**
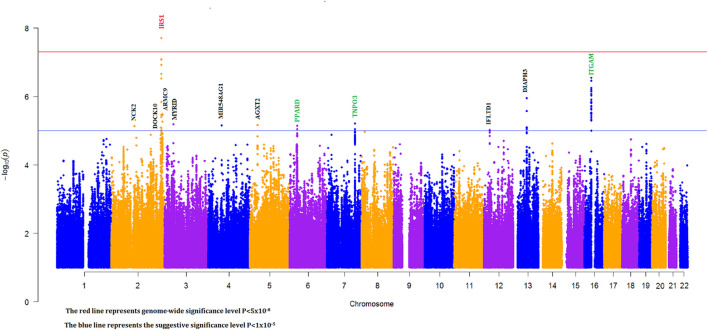
Manhattan plot for imputation-based association with individual ancestry correction (green, black, and red gene names represent known, novel suggestive, and novel genome-wide significant loci, respectively).

To discover novel SLE susceptibility loci and to replicate previously known SLE risk loci in our Egyptian cohort, we performed a genome-wide imputation-based association study with 6,382,276 SNPs while adjusting for individual admixture (see Section 2). The Manhattan plot showing -log_10_ *p* values for SNP associations for our GWAS is shown in Figure 2.

#### 3.2.1 Discovery of novel SLE loci

**FIGURE 3:**
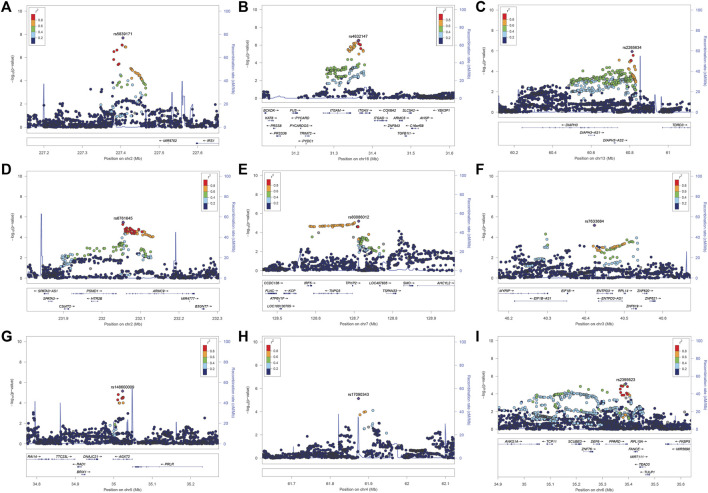
**(A–I)**. LocusZoom plot near the *IRS1/miR-5702* locus, which reached genome-wide significance, and 8 suggestive loci.

We identified 28 SNPs in nine novel loci with *P*_corrected_ < 1 × 10^−5^ (Table 1; Supplementary Table S2; Figure 3). The most significant signal, which passed genome-wise significance (rs5839171, P_corrected_ = 1.98 × 10^−8^, *P*_perm_ = 3.30 × 10^−8^), is downstream of the Insulin receptor substrate 1 gene (*IRS1*) and miR-5702, a microRNA involved in cellular proliferation and cancer risk (Li K. et al., 2019). IRS1 is a signaling adapter protein linking activity of insulin and insulin-like growth factor receptors to intracellular signaling cascades, most notably the PI3K/Akt and Erk MAP kinase pathways (Leslie et al., 2014). *IRS1* has been flagged as a risk locus for insulin resistance (Rung et al., 2009) and type II diabetes (Rung et al., 2009; Voight et al., 2010), and also several cancers (Slattery et al., 2004). The best characterized target of miR-5702 is Zinc finger E-box-binding homeobox 1 (*ZEB1*) (Zhang et al., 2018), a transcription factor critically involved in T-lymphocyte differentiation and interleukin-2 signaling (Guan et al., 2018). At this locus, another nearby (1.7 kb) SNP, rs10804331 (P_corrected_ = 8.5 × 10^−8^), is a significant eQTL for *IRS1* in CD4^+^ naïve T-cells (*p* = 0.002) and CD16^+^ neutrophils (*p* = 0.01) (Supplementary Table S3). This SNP is also a significant mQTL for methylated CpG cg07514207 in the *IRS1* promoter (Chen et al., 2016), potentially modulating transcription.

**TABLE 1:** Association (genome-wide significant and suggestive) signals at the lead SNPs with *p < 1.0x10* ^*−5*^.

Chr	SNP	Position (bp, hg19)	Nearest genes	A1/A2	FA/FU	P_uncorrected	P_corrected	#Sig SNPs	P_perm_admix	OR(95%CI)
2	rs5839171	227,405,634	IRS1/MIR5702	G/GA	0.492/0.597	1.70E-07	1.98E-08	12	3.30E-08	0.63(0.53–0.75)
13	rs2265634	60,812,386	LINC00434, DIAPH3	G/A	0.56/0.47	6.66E-06	1.14E-06	5	3.80E-06	1.49(1.25–1.77)
2	rs6761645	232,055,153	ARMC9	A/T	0.183/0.254	4.06E-06	3.35E-06	4	9.00E-07	0.57(0.45–0.73)
3	rs7633684	40,422,493	ENTPD3, ENTPD3-AS1	T/A	0.803/0.875	1.29E-06	6.66E-06	1	5.80E-06	0.56(0.45–0.71)
5	rs148600009	35,023,218	AGXT2	A/ATTCT	0.201/0.143	4.72E-05	6.88E-06	1	1.31E-05	1.65(1.3–2.1)
4	rs17090343	61,873,612	MIR548AG1	C/T	0.074/0.032	4.33E-06	7.18E-06	1	6.30E-06	2.45(1.67–3.59)
2	rs6743358	106,245,742	NCK2	G/A	0.504/0.411	4.76E-06	7.56E-06	1	6.20E-06	1.48(1.25–1.76)
2	rs11462616	225,900,189	DOCK10	AT/A	0.289/0.377	8.56E-06	8.27E-06	1	1.63E-05	0.67(0.56–0.8)
12	rs12817138	25,826,394	IFLTD1	G/A	0.552/0.638	9.20E-06	9.69E-06	2	7.10E-06	0.67(0.56–0.8)

The second signal (rs2265634; P_corrected_ *=* 1.14 × 10^−6^) is between Diaphanous homolog 1 (*DIAPH3*), which modulates mTOR signaling and cellular proliferation (Wan et al., 2021), and Tudor domain-containing protein 3 (*TDRD3*), a chromatin modulator also involved in proliferation (Morettin et al., 2017); it is also close to long non-coding RNA *LINC00434*. *LINC00434* appears to target several genes involved in cancer and autoimmunity (Supplementary Table S4). This SNP is a significant (*p* = 4.78 × 10^−8^) expression quantitative trait locus (eQTL) for *DIAPH3*, which was a nominally significant (*p* = 7.23 × 10^−3^) SLE risk locus in a previous GWAS study (Lee et al., 2014).

The third signal (rs6761645; P_corrected_ = 3.35 × 10^−6^) is upstream of Armadillo repeat containing 9 [*ARMC9*, encoding KU-MEL-1 (Kiniwa et al., 2001)], a ciliary basal body protein, mutations of which underlie the systemic inflammatory disorder Vogt-Koyanagi-Harada disease (VKH) (Ohno et al., 2019) and the neurodevelopmental disorder Joubert syndrome (Van De Weghe et al., 2017). KU-MEL-1, over-expressed in melanocytes, appears to be a primary autoantigen in VKH (Otani et al., 2006) and is also associated with vitiligo and autoimmune uveitis. These top three hits were all located in active chromatin, with rs6761645, in particular, overlapping promoter signals in 17 tissues and enhancers in 12 tissues (Ward and Kellis, 2012); having 14 significant eQTL hits (Supplementary Table S3); and being associated with transcription factor binding sites for Hypoxia-inducible factor 1 (HIF1), a critical regulator of inflammation and the DNA damage response (Dehne and Brüne, 2009; Ramachandran et al., 2021). rs6761645 is an eQTL for *PSMD1* (26S proteasome non-ATPase regulatory subunit 1) (Supplementary Table S3), another proliferation marker (Okumura et al., 2017).

The five other novel signals localized to *ENTPD3/ENTPD3-AS1* (a tumor suppressor (Li M. et al., 2019) that strongly signals through HIF1 (Wang J. et al., 2021) and its associated antisense RNA)*, NCK2* [a critical immune adaptor protein linking B-cell receptor activation to PI3K signaling (Cannons et al., 2013)], *IFLTD1* [a ciliary organization protein and cancer risk gene (Wang et al., 2005), also known as *LMNTD1* and *PAS1C1*], *AGXT2* [a risk locus of premature myocardial infarct (Yuanfeng et al., 2016)], *DOCK10* [critical in B-cell activation and proliferation (Yelo et al., 2008)], and the microRNA *MIR548AG1*.

Given the prominent role played by microRNAs in cancer (Calin and Croce, 2006) and autoimmunity (Xiao and Rajewsky, 2009), and the fact that miR-548ag1 had the largest effect size of our novel loci [odds ratio = 2.45 (95% confidence interval 1.67–3.59)], we closely examined the apparent involvement of this miRNA in SLE. The miR-548 family was first discovered in colorectal expression arrays (Cummins et al., 2006) and was expanded through bioinformatic searches for miRNAs overlapping repetitive elements (Piriyapongsa and Jordan, 2007). Both the microRNA family itself and putative target sites derive from the Made1 repetitive element; these sequences form very stable hairpin structures of ∼37 bp in length. miR-548ag1 was discovered from a B-cell tumor line (Jima et al., 2010), suggesting immune involvement. Many other family members are implicated in cancer (Shi et al., 2015; Ke et al., 2016) and autoimmunity (Yu et al., 2017; Cakmak Genc et al., 2018; Li et al., 2018). To further elucidate the biological roles of miR-548ag1, we searched the human genome for miR-548ag1-like sequences (see Section 2), restricting ourselves to loci exhibiting primate-specific insertions [consistent with other verified miR-548 family members (Piriyapongsa and Jordan, 2007)]. We found closely related sequences in UTRs and introns of ten genes—usually overlapping enhancers and/or transcription factor binding sites (Supplementary Table S5). Strikingly, all ten genes have clear immune involvement (Supplementary Table S5). Five of the ten hits are expressed sequence tags (ESTs) of ∼90 bp–100 bp and almost certainly represent previously unannotated members of the miR-548 family. Of the remaining five, it is not immediately clear which are microRNAs themselves and which are potential miRNA target sites. Together, these results emphasize the immune system relevance of our SLE risk locus miR-548ag1 and related miR-548 family members.

#### 3.2.2 Replication of known SLE loci

We compared our association results with 179 SLE known loci (see Section 2) and found 97 known loci replicated with at least one significantly (P_perm_ < 0.01) associated SNP. Whenever available, we showed LD with the top published variant to identify independent associations at known loci (Table 2). The most significant SNPs at each locus are listed in Table 2. Among them, prominent (P_perm_ < 5.0 × 10^−5^) signals include *ITGAM-ITGAX* (rs71391210; P_perm_ = 2.0 × 10^−7^), *IRF5* (rs80086012; P_perm_ = 3.5 × 10^−6^), *DEF6-PPARD* (rs2395623; P_perm_ = 4.70 × 10^−6^), *XKR6* (rs2409660; P_perm_ = 4.55 × 10^−5^), *IRF1* (rs10586626; P_perm_ = 5.43 × 10^−5^), and *TYK2* (rs77389625; P_perm_ = 1.91 × 10^−4^). All replicated (P_perm_ < 0.01) SNPs and their nearest genes are shown in Table 2.

**TABLE 2:** Replication of 97 published loci at *P*_*perm*_<0.01.

Chr	Published_ SNP	PMID	Top_SNP	Nearest genes	*r* ^2^_pub _top	A1	F_A	F_U	A2	P_uncorr_	P_admix_	P_perm_	#Sig_SNP	OR
16	rs34572943	28714469	rs71391210	ITGAM, ITGAX	0.40	T	0.38	0.284	C	8.97E-07	3.91E-07	2.00E-07	170	1.56
7	rs3757387	33272962	rs80086012	IRF5, TNPO3	0.06	T	0.166	0.11	C	3.00E-05	1.37E-05	3.50E-06	101	1.72
6	rs10807150	26808113	rs2395623	ANKS1A, PPARD, UHRF1BP1, DEF6	0.40	C	0.466	0.567	T	1.22E-06	3.65E-06	3.70E-06	205	0.66
8	rs7819602	28714469	rs2409660	AC011008.2, XKR6	0.09	A	0.235	0.168	G	5.77E-05	5.10E-05	4.55E-05	44	1.52
19	rs12461589	33272962	rs68013007	ANKRD27, PDCD5	0.01	T	0.203	0.144	A	4.62E-05	2.78E-05	4.69E-05	24	1.63
2	rs9630991	33536424	rs59207796	AC108047.1	0.56	C	0.206	0.273	CACATG	7.03E-05	1.10E-04	5.34E-05	110	0.65
5	rs2549002	33272962	5:131813034	IRF1	0.30	A	0.267	0.203	AAAG	1.15E-04	5.99E-05	5.43E-05	92	1.5
6	rs35789010	28714469	rs1321248	CARMIL1	0.01	C	0.2	0.267	T	1.32E-04	1.11E-04	6.97E-05	13	0.67
6	rs148314165	33272962	rs374184737	BTF3L4P3, LINC02528, TNFAIP3	0.04	T	0.476	0.569	TAA	1.20E-05	5.14E-05	7.48E-05	59	0.69
1	rs4844538	33272962	rs28584674	IKBKE, IL10, IL19, SRGAP2	0.00	C	0.081	0.129	T	3.17E-05	2.77E-05	1.42E-04	13	0.5
19	rs55882956	33272962	rs77389625	TYK2	mono	A	0.042	0.075	G	2.98E-04	2.78E-04	1.91E-04	16	0.44
8	rs16902895	33272962	rs6470627	LINC00824	0.63	G	0.848	0.898	T	1.63E-04	5.32E-04	2.92E-04	70	0.61
7	rs4598207	33272962	rs4917016	C7orf72, IKZF1	0.06	C	0.722	0.779	T	1.95E-04	2.76E-04	2.94E-04	5	0.66
1	rs2205960	33272962	rs34697014	LOC100506023, TNFSF4	0.00	G	0.251	0.312	A	6.16E-04	3.80E-04	3.77E-04	85	0.71
10	rs111447985	33272962	rs1008463	STN1	0.00	G	0.864	0.907	A	1.87E-04	4.15E-04	3.80E-04	9	0.57
5	rs2431697	33272962	rs1422980	MIR3142, MIR3142HG	0.03	T	0.368	0.3	C	4.82E-04	5.96E-04	4.06E-04	28	1.37
19	rs4801882	33272962	rs11672086	SIGLEC5	0.02	C	0.289	0.228	T	2.53E-04	1.46E-04	4.78E-04	55	1.46
13	rs1885889	33536424	rs11619751	AL136961.1, TM9SF2	0.47	A	0.136	0.094	G	1.04E-03	7.53E-04	5.24E-04	15	1.56
11	rs10896045	33272962	rs36089663	AP5B1, OVOL1	0.01	TA	0.296	0.237	T	5.24E-04	6.23E-04	5.85E-04	14	1.42
13	rs76725306	33536424	rs10675447	AL135901.1, RCBTB1	0.00	TTA	0.749	0.686	T	3.30E-04	3.21E-04	6.08E-04	104	1.45
11	rs77885959	33272962	rs4150658	GTF2H1	mono	G	0.144	0.113	A	1.08E-02	1.97E-03	6.36E-04	2	1.45
1	rs3806357	33272962	rs72744822	ELF3	0.00	A	0.136	0.096	G	1.25E-03	8.35E-04	6.46E-04	32	1.58
12	rs77465633	33272962	rs66480035	ATXN2	mono	C	0.463	0.524	T	6.80E-04	3.65E-04	6.85E-04	80	0.71
1	rs76107698	33272962	rs140778333	AL590385.2, FCGR2A, FCGR2C	0.00	T	0.022	0.049	C	8.21E-04	9.38E-04	6.99E-04	10	0.42
9	rs1887428	33272962	rs33973400	JAK2	0.00	C	0.371	0.441	CT	2.08E-04	2.14E-04	7.67E-04	17	0.71
17	rs2671655	33272962	rs140070508	LOC102724596	0.00	G	0.044	0.022	A	1.85E-03	2.81E-03	7.79E-04	30	2.16
22	rs4819670	33272962	rs5993014	USP18	0.25	A	0.3	0.242	G	9.45E-04	9.02E-04	8.56E-04	3	1.39
4	rs6841907	33272962	rs1129617	COQ2	0.21	A	0.225	0.284	G	4.16E-04	6.94E-04	8.60E-04	42	0.68
10	rs7097397	33272962	rs2620895	LRRC18, PCDH15, WDFY4	0.04	A	0.121	0.076	G	1.63E-04	8.47E-04	8.83E-04	3	1.74
8	rs2428	29625966	rs117234614	MFHAS1	0.03	C	0.064	0.043	T	7.76E-03	3.19E-03	9.56E-04	32	1.75
2	rs3087243	33536424	rs11374410	CTLA4, ICOS	0.00	GA	0.127	0.088	G	1.03E-03	6.19E-04	1.01E-03	19	1.6
1	rs1547624	33536424	rs10921148	AL390957.1	0.01	T	0.195	0.147	C	4.53E-04	3.74E-04	1.02E-03	6	1.56
12	rs6539078	33272962	rs703619	AC084364.4, LOC105369945	0.00	A	0.373	0.439	G	1.42E-03	1.63E-03	1.09E-03	8	0.76
17	rs2286672	26502338	rs3786042	PLD2	0.98	T	0.104	0.068	C	3.10E-04	3.36E-04	1.10E-03	4	1.87
1	rs1780813	29848360	rs142583842	SMYD3	0.65	ATAGC	0.031	0.057	A	2.66E-03	2.09E-03	1.16E-03	52	0.5
5	rs2421184	33272962	rs17056704	LINC01845	0.02	C	0.217	0.169	G	1.48E-03	1.95E-03	1.19E-03	38	1.44
16	rs11117432	33272962	rs11117444	AC092723, IRF8	0.00	C	0.219	0.272	G	9.96E-04	6.20E-04	1.22E-03	25	0.69
12	rs2540119	33272962	rs142451408	PARP11	0.09	A	0.504	0.562	AAAG	2.40E-03	1.65E-03	1.23E-03	16	0.76
15	rs35985016	33272962	rs1455854	LRRK1	0.00	T	0.378	0.319	C	1.01E-03	6.47E-04	1.35E-03	24	1.38
14	rs12148050	33536424	rs4900554	TRAF3	0.02	A	0.122	0.153	G	1.42E-02	7.56E-03	1.36E-03	3	0.7
19	rs10419308	33536424	rs7259964	AC010327, TMEM86B	0.00	G	0.133	0.088	T	3.91E-04	1.08E-03	1.37E-03	3	1.63
6	rs597325	33272962	rs13209535	BACH2	0.00	G	0.122	0.091	A	9.83E-03	1.99E-03	1.56E-03	14	1.46
3	rs7637844	33272962	rs6779548	LINC00870	0.03	G	0.656	0.718	A	1.66E-03	1.50E-03	1.56E-03	12	0.76
6	rs36014129	28714469	rs77341667	H2AC3P, H2BP5	0.01	T	0.032	0.056	C	5.68E-03	3.49E-03	1.57E-03	2	0.53
12	rs200521476	33272962	rs11610045	FBRSL1	0.11	A	0.361	0.428	G	1.12E-03	2.18E-03	1.63E-03	34	0.75
2	rs11889341	33272962	rs11677408	STAT4	0.02	T	0.063	0.04	C	4.33E-03	2.66E-03	1.68E-03	3	1.83
1	rs12093154	33536424	rs1240748	C1QTNF12	0.08	T	0.708	0.657	C	7.85E-03	1.92E-03	1.72E-03	9	1.29
13	rs57141708	33272962	rs9566681	ELF1	0.20	T	0.11	0.077	C	2.60E-03	4.37E-03	1.80E-03	1	1.61
11	rs2785198	33272962	11:35203468	LOC100507144, PDHX	0.01	TTA	0.418	0.476	T	2.51E-03	1.84E-03	1.90E-03	11	0.76
12	rs11059928	33272962	rs5801815	SLC15A4	0.00	C	0.352	0.406	CA	2.32E-03	2.92E-03	2.08E-03	4	0.73
18	rs1788097	33272962	rs34594414	CD226	0.24	T	0.381	0.322	A	6.89E-04	1.21E-03	2.25E-03	4	1.41
10	rs77448389	33272962	rs2246268	ANKRD16	0.02	C	0.065	0.04	A	1.82E-03	2.32E-03	2.42E-03	7	1.96
12	rs4622329	33272962	rs144176212	DRAM1	0.04	C	0.707	0.763	CTTTT	6.63E-04	4.10E-04	2.49E-03	23	0.7
11	rs4930642	33272962	rs2924520	TPCN2	0.04	G	0.378	0.442	A	1.17E-03	1.82E-03	2.53E-03	30	0.74
8	rs17374162	33272962	rs2099950	AS1, MSC	0.04	C	0.203	0.159	G	4.58E-03	3.82E-03	2.55E-03	13	1.37
19	rs7251	32719713	rs8103298	IRF3	0.02	C	0.056	0.087	T	5.48E-03	3.73E-03	2.58E-03	4	0.63
10	rs10823829	33272962	rs7909048	CDH23	0.05	T	0.066	0.038	G	1.43E-03	3.24E-03	2.74E-03	7	1.86
22	rs4821116	33272962	rs4820091	CCDC116, UBE2L3, YDJC	0.57	G	0.349	0.289	T	1.53E-03	2.09E-03	2.77E-03	67	1.33
4	rs13101828	33272962	rs41286651	DGKQ	0.04	C	0.043	0.022	T	5.18E-03	5.06E-03	3.02E-03	1	1.94
3	rs6762714	27399966	rs1152846	LPP	0.19	C	0.664	0.718	T	5.40E-03	4.28E-03	3.03E-03	13	0.78
10	rs58164562	33272962	rs2031130	BBIP1	0.01	G	0.588	0.644	T	6.03E-03	4.49E-03	3.05E-03	4	0.79
1	rs2476601	26502338	rs6663888	PHTF1, PTPN22, RSBN1	0.00	A	0.035	0.051	C	2.71E-02	5.90E-03	3.07E-03	3	0.56
5	rs10036748	33272962	rs960709	TNIP1	0.96	G	0.425	0.36	A	1.21E-03	2.53E-03	3.15E-03	6	1.32
14	rs2819426	33272962	rs33925946	AHNAK2, AHNAK2, PLD4	0.04	T	0.176	0.215	G	9.39E-03	4.64E-03	3.23E-03	5	0.73
16	rs11288784	33272962	rs11076521	HEATR3	0.11	T	0.628	0.582	C	8.48E-03	3.79E-03	3.46E-03	10	1.3
2	rs13385731	33272962	rs4569454	RASGRP3	0.00	G	0.628	0.69	A	9.61E-04	1.02E-03	3.80E-03	2	0.74
1	rs13306575	33272962	rs17849502	NCF2, NMNAT2, SMG7	mono	T	0.072	0.044	G	5.72E-03	5.81E-03	3.81E-03	2	1.61
17	rs8072449	28714469	rs147427721	AC011933.4, GRB2, SLC25A19	0.15	T	0.876	0.849	TTC	4.29E-02	1.06E-02	3.90E-03	6	1.31
1	rs116785379	33272962	1:157081831	ETV3	0.00	G	0.075	0.05	C	4.30E-03	5.79E-03	3.92E-03	1	1.73
6	rs9488914	33272962	rs1204826	DSE	0.29	G	0.665	0.708	A	1.88E-02	1.03E-02	4.12E-03	4	0.8
18	rs118075465	33272962	rs34787266	LOC284241	0.00	T	0.094	0.129	C	9.59E-03	5.27E-03	4.38E-03	10	0.7
3	rs564976	26502338	rs7652547	AS1, IL12A	0.01	C	0.198	0.247	T	6.09E-03	5.10E-03	4.46E-03	7	0.76
4	rs231694	33272962	rs116412781	FAM193A, TNIP2	0.15	T	0.078	0.047	C	9.11E-04	6.70E-04	4.53E-03	3	1.84
16	rs11376510	33272962	rs889791	MAFTRR	0.00	T	0.098	0.127	G	1.37E-02	8.79E-03	4.59E-03	5	0.68
17	rs35966917	33272962	rs79185281	TNFRSF13B	0.10	C	0.542	0.589	CT	1.02E-02	1.26E-02	4.61E-03	0	0.78
6	rs9322454	33272962	rs12199124	IPCEF1	0.14	A	0.066	0.042	G	7.21E-03	7.41E-03	4.69E-03	0	1.67
1	rs3795310	33536424	rs72635735	RERE	0.00	C	0.052	0.032	T	6.19E-03	8.01E-03	4.71E-03	1	1.89
4	rs2855772	29494758	rs2646326	KIT	0.00	A	0.068	0.046	G	1.44E-02	8.59E-03	5.32E-03	0	1.6
11	rs9736939	33272962	rs12792061	AP001122.1, ETS1, LINC02098	0.02	A	0.112	0.152	G	4.76E-03	7.73E-03	5.82E-03	4	0.69
1	rs11264750	33536424	rs10796979	FCRL5	0.20	C	0.854	0.811	A	6.49E-03	4.26E-03	6.20E-03	16	1.37
3	rs9852465	28714469	rs7638793	AC116036.2, PDHB, PXK	0.05	C	0.274	0.224	T	2.77E-03	4.33E-03	6.22E-03	27	1.36
5	rs6871748	33536424	rs79041667	AC112204.3, IL7R	0.01	G	0.193	0.149	A	3.85E-03	3.51E-03	6.28E-03	8	1.39
1	rs28411034	33536424	rs34755028	MTF1	0.06	C	0.671	0.721	CA	6.10E-03	7.74E-03	6.75E-03	1	0.77
3	rs144104218	33272962	rs20568	CD80, TIMMDC1, TMEM39A	0.01	T	0.098	0.068	C	1.07E-02	7.91E-03	6.82E-03	3	1.47
1	rs6702599	33536424	rs35189936	IL12RB2	0.00	A	0.076	0.112	AT	2.41E-03	3.92E-03	7.25E-03	19	0.61
8	rs2736332	33272962	8:11346080	AF131216.5, BLK	0.13	G	0.117	0.083	T	1.93E-03	2.56E-03	7.37E-03	4	1.62
10	rs7902146	33272962	rs76465877	ARID5B	0.00	A	0.048	0.028	G	6.67E-03	7.34E-03	7.41E-03	1	1.84
17	rs61759532	33272962	rs7214863	AC026954.1, ACAP1	0.00	T	0.629	0.591	C	3.21E-02	1.16E-02	7.52E-03	1	1.23
7	rs117026326	33272962	rs10256306	GTF2IRD1, LOC101926943	0.01	A	0.072	0.049	G	1.95E-02	9.88E-03	7.53E-03	1	1.5
14	rs911263	28714469	rs17828548	RAD51B	0.01	T	0.047	0.07	C	1.03E-02	1.21E-02	7.90E-03	0	0.59
4	rs58107865	33272962	rs10516550	LEF1	mono	T	0.047	0.069	C	8.88E-03	6.80E-03	7.92E-03	2	0.55
5	rs7725218	33272962	rs72715511	TERT	0.00	T	0.223	0.186	C	1.92E-02	6.28E-03	8.22E-03	1	1.3
15	rs8023715	24871463	rs79487017	LINC02253, RN7SKP181	0.00	A	0.03	0.058	G	2.08E-03	3.92E-03	8.43E-03	3	0.49
12	rs4251697	33272962	rs116902872	CDKN1B, CREBL2, GPR19	0.00	A	0.025	0.045	G	1.25E-02	1.07E-02	8.74E-03	0	0.54
2	rs2381401	33536424	rs10048784	ARHGAP15	0.01	C	0.707	0.662	T	2.36E-02	1.33E-02	8.99E-03	3	1.22
4	rs10018951	29494758	4:184474355	TRAPPC11	0.00	T	0.913	0.888	C	2.43E-02	2.48E-02	9.18E-03	0	1.45
4	rs4643809	33272962	rs79879350	BANK1	0.09	T	0.046	0.07	C	5.69E-03	2.56E-03	9.35E-03	8	0.53
8	rs2953898	28714469	rs115578289	RPS20	0.01	C	0.044	0.026	T	1.53E-02	1.24E-02	9.37E-03	1	1.78
mono = Monomorphic SNP														

#### 3.2.3 Minor allele frequency of associated SNPs and ethnicity

**FIGURE 4:**
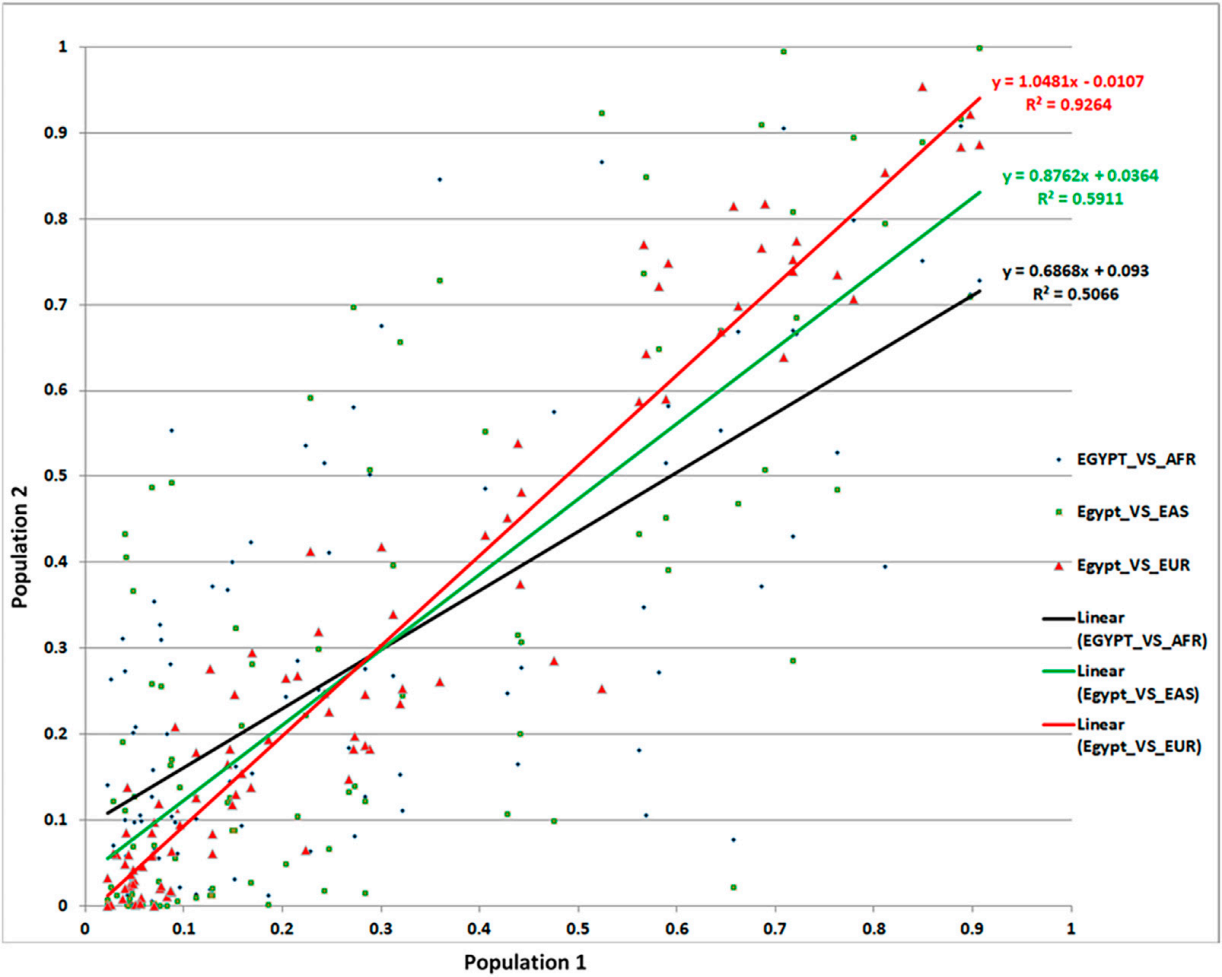
Comparison of MAFs for the associated SNPs from our Egyptian controls and 1,000 Genomes data.

Allelic abundance—most often reported as minor allele frequency (MAF)—is an important measure both to estimate the effect of given SNPs on specific populations and more broadly to compare ancestry and population stratification between diverse populations. Using the 97 total replicated lead SNPs, we compared MAFs in our Egyptian cohort with those of Africans, Europeans, and Asians. We found a remarkable similarity between the MAFs of Egyptians and Europeans (*R* ^2^ = 93%)—i.e., the MAF variance in Egyptians was well explained by the MAF variance in Europeans. Conversely, correlation with Africans (*R* ^2^ = 51%) and Asians (*R* ^2^ = 59%) was markedly lower (Figure 4). Although surprising *prima facie*, these results were supported by our admixture analysis (Supplementary Figure S3).

#### 3.2.4 Functional SNPs from some notable loci

**FIGURE 5:**
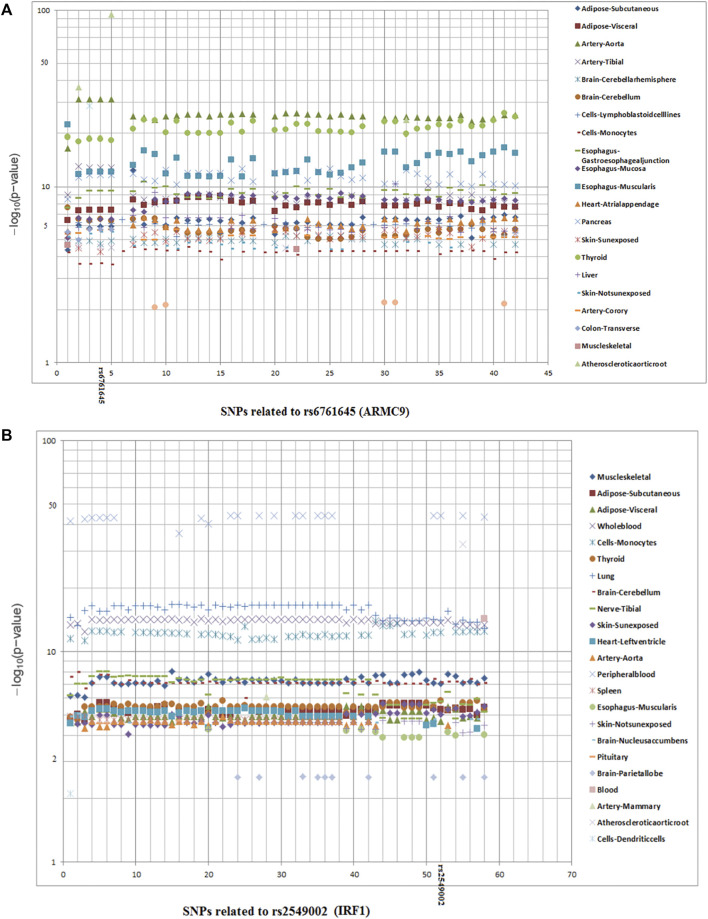
**(A,B)**. eQTL plots for the related SNPs (*r* ^2^ > 0.8 with top SNPs) in two notable loci.

Having identified several new SLE-associated loci, most of which have known roles in both immune homeostasis and other autoimmune disorders, we sought to better establish possible mechanisms for both the loci and the associated SNPs. For our nine novel loci, we first looked for significant (*P* < 1 × 10^−5^) eQTLs for both the top SNPs and the larger set of SNPs in linkage disequilibrium (*r* ^2^ > 0.8) with them. We found several significant (3.5 × 10^−95^ < *P* < 1 × 10^−2^) eQTLs from our new loci, particularly from *ARMC9* (43 SNPs; 25 tissues), *IFLTD1* (5 SNPs; 4 tissues), *DIAPH3* (6 SNPs; 1 tissue), and *ENTPD3* (1 SNP; 10 tissues) (Figure 5A). *IFLTD1* is enriched in the brain, sex organs, and digestive tract (https://www.proteinatlas.org), and most eQTL signals came from these tissues. *ARMC9* is very strongly expressed in natural killer (NK) cells (https://www.proteinatlas.org) and melanocytes (Otani et al., 2006); interestingly, the strongest eQTL signals arose from vascular endothelium and thyroid. Known loci also contained many eQTL SNPs; for example, *IRF1* (58 SNPs; 23 tissues) showed particularly strong eQTL signals. *IRF1* is quite immune cell-specific, and accordingly, the strongest eQTL signals came from blood (Figure 5B).

## 4 Discussion

Our study represents the first lupus GWAS in North Africans, an underrepresented population in human genetic studies. Previous studies indicated that Egyptians are highly admixed, with substantial ancestry arising from the Middle East, Europe, and Africa—with the extent of European and Caucasus hunter-gatherer (Lazaridis et al., 2016) contribution being higher, and the African contribution being lower, than might be expected from geography. Greater European/Middle Eastern than African ancestry is also supported by whole-genome sequencing (ElHefnawi et al., 2021). Importantly, modern Egyptians are quite divergent from ancient populations. The inclusion of more diverse participants in genomics studies has been shown to significantly increase fine-mapping resolution (Zaitlen et al., 2010; Asimit et al., 2016). Further studies in Egyptians (and other North African and Middle Eastern populations) could potentially reveal more genetic associations, with implications for understanding, diagnosing, and treating disease for Egyptians and all humans.

In this study, we identified nine novel loci; one is genome-wide significant for lupus susceptibility and eight are suggestive. Among 179 known non-HLA SLE susceptibility loci, 97 were successfully replicated—indicating that most lupus susceptibility in Egyptians is ancestry-independent. We also found several loci not previously reported in any populations. Intriguingly, the novel loci were substantially enriched in proteins involved in cellular proliferation and cancer risk. Specifically, Insulin receptor substrate 1 (*IRS1*), the microRNA miR-5702, Diaphanous homolog 1 (*DIAPH3*), Armadillo repeat containing 9 (*ARMC9*, *a.k.a.* KU-MEL-1), Intermediate filament tail domain containing 1 (*IFLTD1*, *a.k.a. LMNTD1*), immune adaptor protein NCK2 (Labelle-Côté et al., 2011), and Ectonucleoside triphosphate diphosphohydrolase 3 (*ENTPD3*) (Wang J. et al., 2021) all have well-documented roles in cellular proliferation and tumor invasion. Proliferation factors play a prominent role in lupus, particularly in invasion of specific organs in sub-clinical phenotypes, most notably lupus nephritis (Balomenos et al., 2000; Treamtrakanpon et al., 2012).

The novel and replicated loci in this study share other features in addition to association with cellular proliferation—for instance, regulation of and by microRNAs, another hallmark of SLE (Dai et al., 2007; Carlsen et al., 2013). Notably, both *IRS1* and *ARMC9* (along with *LRIG2*, *PSPH*, and *SKP2*) are direct targets down-regulated by miRNA-150 (Zhang et al., 2021). miRNA-150 is a critical immune regulator, for instance driving expression of the inflammatory receptor TREM-1 (Triggering receptor expressed on myeloid cells 1) in dendritic cells and contributing to lupus-like symptoms in mice (Gao et al., 2017). More recently, miRNA-150 (along with miRNA-148b) has been proposed as a specific biomarker for lupus nephritis (Alkhateeb and Altamemi, 2021). Two of our novel risk loci even interact at the protein level: NCK2 directly binds to IRS1 to create an intracellular signal transduction mechanism for signaling through insulin and insulin-like growth factor receptors (Tu et al., 1998; Tu et al., 2001). We performed a sequence search with our novel risk miRNA *miR548AG1* and discovered ten closely related sequences, all of them in introns or UTRs of immune-critical genes (Supplementary Table S5) –many overlapping enhancers and/or transcription factor binding sites. Furthermore, five of these ten hits are ESTs of ∼90 bp–100 bp and almost certainly constitute previously unannotated members of the miR-548 family. The clear immune association of all ten closely related sequences further confirms the relevance of our SLE risk locus *miR548AG1*.

Our study very carefully controlled for admixture in estimating effect size and significance level. This was a critical step, as multiple loci became more significant or less significant once controlled for admixture (Tables 1, 2; Supplementary Table S1). The most substantive change was at the novel locus *IRS1/miR-5702,* where the top SNP rs5839171 achieved genome-wide significance after admixture correction (*P*_uncorrected_ = 1.70 × 10^−7^, P_corrected_ = 1.98 × 10^−8^). This remains significant (P_permuted_ = 3.3 × 10^−8^) after 10^9^ permutations. This emphasizes the utility of admixed populations, like Egyptians, in studying novel gene discovery.

To assess the replication of previously known loci [Table 1 from (Ha et al., 2022)], we implemented a very large (10^7^–10^9^ combinations) ancestry-adjusted permutation analysis on our data. Critically, permutation analysis controls for low sample-size effects, as all permutations have identical *n* as the proposal—comparisons are between permutations with identical statistical power *a priori* to produce an association. Using the ancestry-corrected permutation-based analysis, we set threshold for replication at *p* < 0.01. Here we further explain our reasoning behind the robustness of our permutation analysis and some of its findings.

First, a recent large-scale (*n* > 208,000) SLE GWAS meta-analysis on East-Asian populations identified rs956237 (GWAS lead variant; intron of *LEF1*, Lymphoid enhancer-binding factor 1—a leukocyte transcription factor) at 4q25 as a novel susceptibility locus (Yin et al., 2020; *p* < 4.0 × 10^−11^). Reassuringly, a companion transcriptome-wide association study (TWAS) using data from Asian samples also strongly indicated *LEF1* as the primary gene underlying 4q25 association (Yin et al., 2020; *p* < 1.3 × 10^−10^). However, our primary reference (Table 1 from Ha et al., 2022) nominated rs58107865 instead of rs956237 as lead SNP. In our study, we found that rs58107865 does not exist in the African/Egyptian population. Instead, we discovered an association with another intronic *LEF1* SNP (rs10516550, *p* = 0.008), common in African/European/Hispanic populations (but not Asian). Of course, this SNP needs to be independently replicated in a population with similar ethnic background. But we believe that it’s appropriate to cautiously interpret this in the replication category.

Second, *NCF2* is a very well-known SLE locus, where multiple studies (Cunninghame Graham et al., 2011; Yin et al., 2020) including ours (Kim-Howard et al., 2014) have reported non-coding and at least 3 independent ethnicity-specific coding variants within *NCF2* passing genome-wide significance in Asian/Hispanic (rs13306575), European/African-American (rs17849502) and African-American (rs35937854) populations. This locus was represented by rs13306575 in our reference data (Table 1 from Ha et al., 2022). In our data, we found evidence of association (ancestry-adjusted permutation *p* = 0.004) with nearby (< 150 bp) rs17849502. Thus, we have found essentially the same result as the prior studies; however, this real association falls below the strict threshold set by the Bonferroni correction (*p =* 0.05/179 = 0.00027). Our non-parametric permutation analysis, however, detects this locus as a significant association, which we believe is supported by the preponderance of evidence.

Although the sample sizes in the current study are not overly large, we had the power to attain multiple noteworthy discoveries for several reasons: 1) The highly admixed Egyptian population gave us the power to discover nine novel loci, one of which achieved genome-wide significance. 2) Our data attained a remarkable replication rate (97 out of 179 loci; 54%)—increasing confidence in both known and novel loci, and supporting the notion that despite different genetic backgrounds, underlying loci and risk alleles are broadly conserved across ethnicities (Marigorta and Navarro, 2013). Over 80% of our replicated loci arose from GWASs on Europeans, indicating broad concordance of genetic risk. However, it is important to note that the studies included in Ha et al. (2022) were performed on populations with large sample sizes and statistical power. Egyptians have a stronger contribution from Middle Eastern ancestries than European (Wohlers et al., 2020), and their population history and admixture proportions are different. Moreover, our sample size was relatively small. Future studies will boost sample sizes and further address the other aspects. 3) We found several common pathways shared by the known and new loci, increasing the robustness of the observations, and laying out the foundation for further experimental studies to establish a clear mechanistic basis for these loci contributing to SLE risk and progression.

However, we also acknowledge that lack of an independent replication, especially for the novel associations, is a limitation of this study. Therefore, in addition to strengthening the observations and establishing mechanisms, further studies with larger sample sizes will clearly delineate the ancestry-independent and ancestry-specific components of SLE association.

**Institutional Review Board Statement:** The study was conducted in accordance with the Declaration of Helsinki and approved by the Institutional Review Board of the Oklahoma Medical Research Foundation (IRB approval number 14–16), and Mansoura University (Egypt) Ethics committee/IRB: MFM-IRB code number R/16.04.81.

**Informed Consent Statement:** Informed consent was obtained from all subjects involved in the study.

## Data Availability

The original contributions presented in the study are included in the article/Supplementary Materials, further inquiries can be directed to the corresponding authors.
