# Supplementary material for: Genome-wide association study for systemic lupus erythematosus in an egyptian population
Source: Front Genet. 2022 Oct 17;13:948505. doi: 10.3389/fgene.2022.948505 (PMC9619055; doi:10.3389/fgene.2022.948505)
Supplement: Supplementary file 1 [file DataSheet1.docx]

**SUPPLEMENTARY FIGURES**


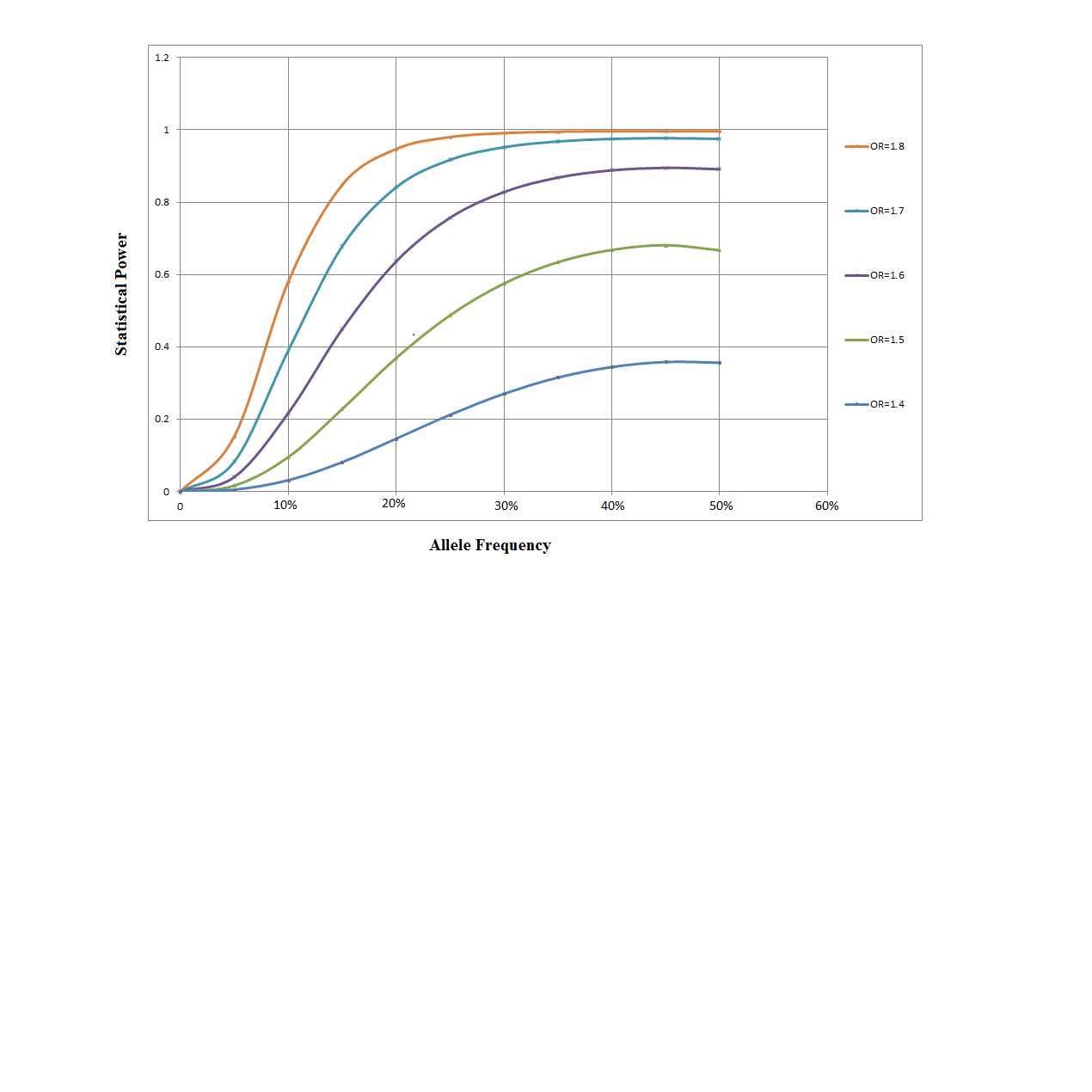
**Supplementary Figure 1.** Statistical power under different MAFs (in proportion) and ORs at α=1x10^-5^.

**Supplementary Figure 2.** QQ plot for the imputed dataset in our study.


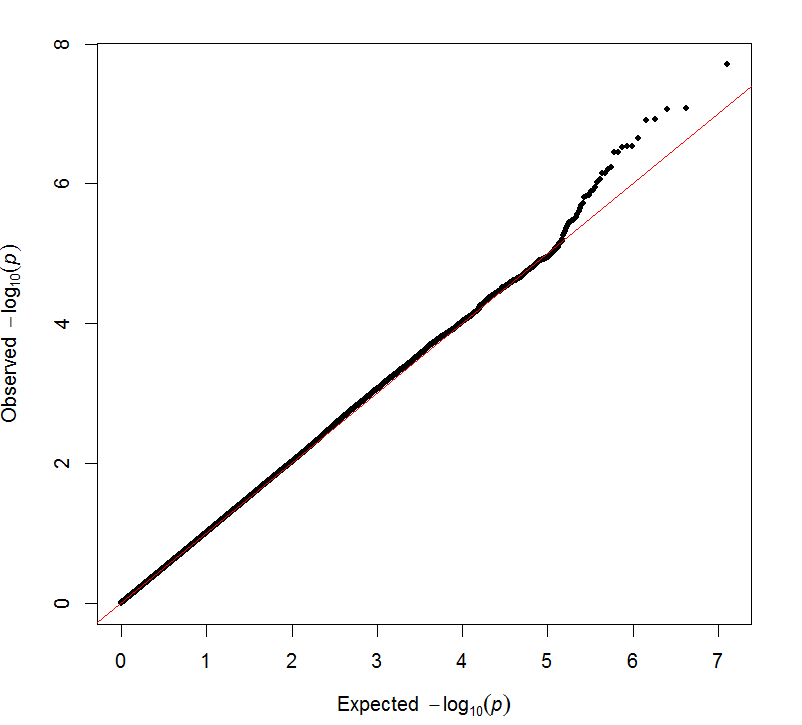


**Supplementary Figure 3.** Principal components analysis of the samples (before and after clean up) as well as together with Near Eastern data.


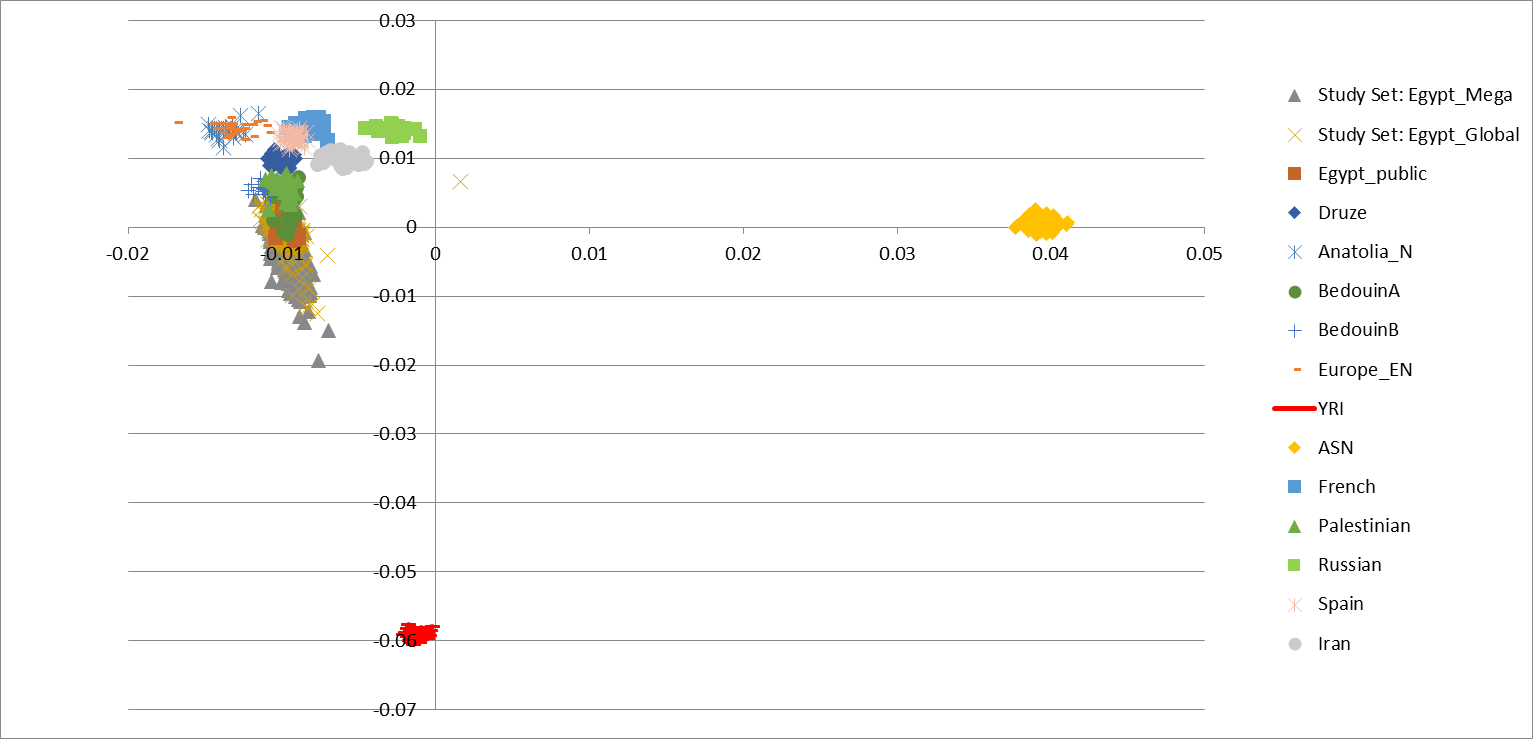


**PC1 VS PC2**

**PC1 VS PC3**


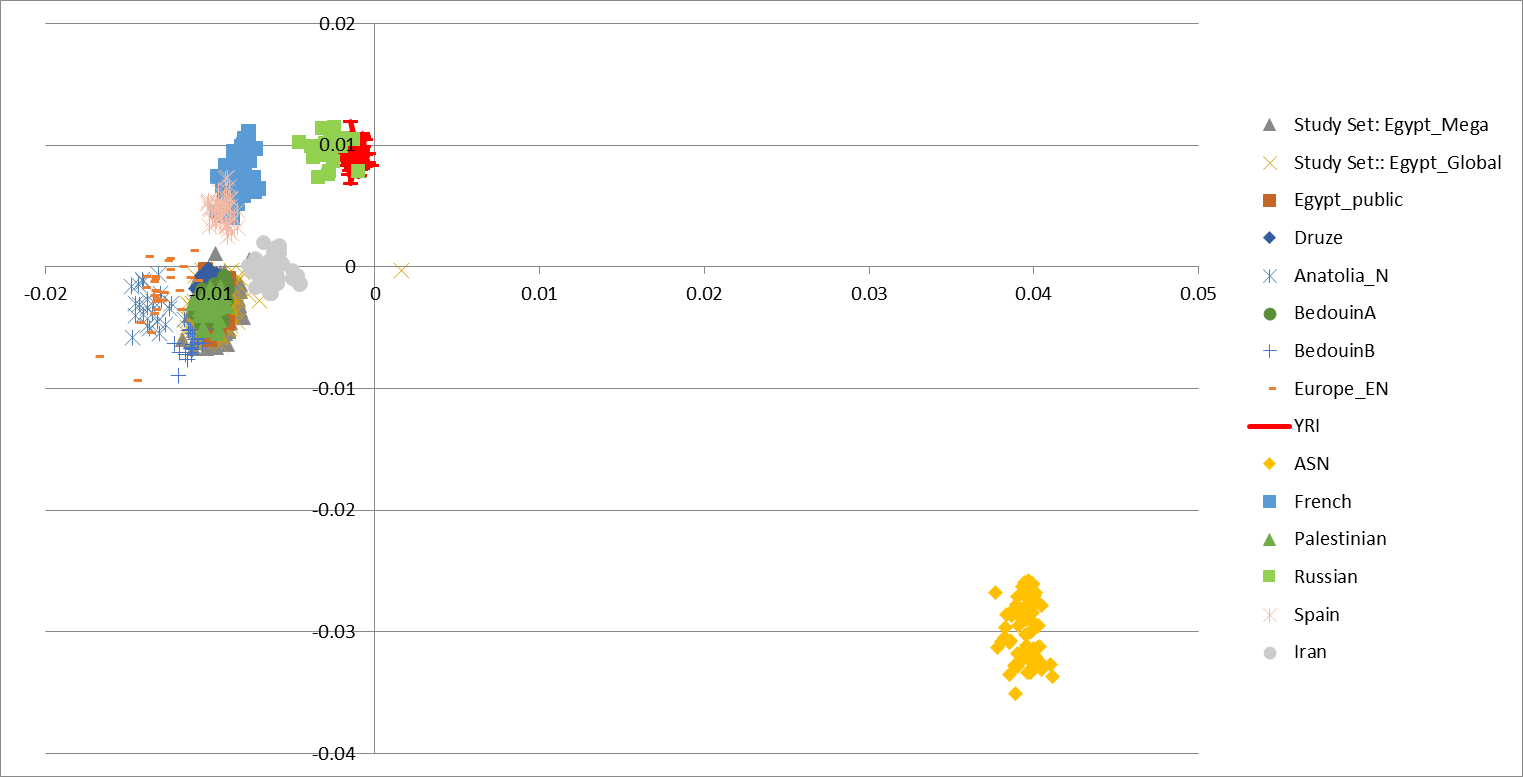


**PC2 VS PC3**


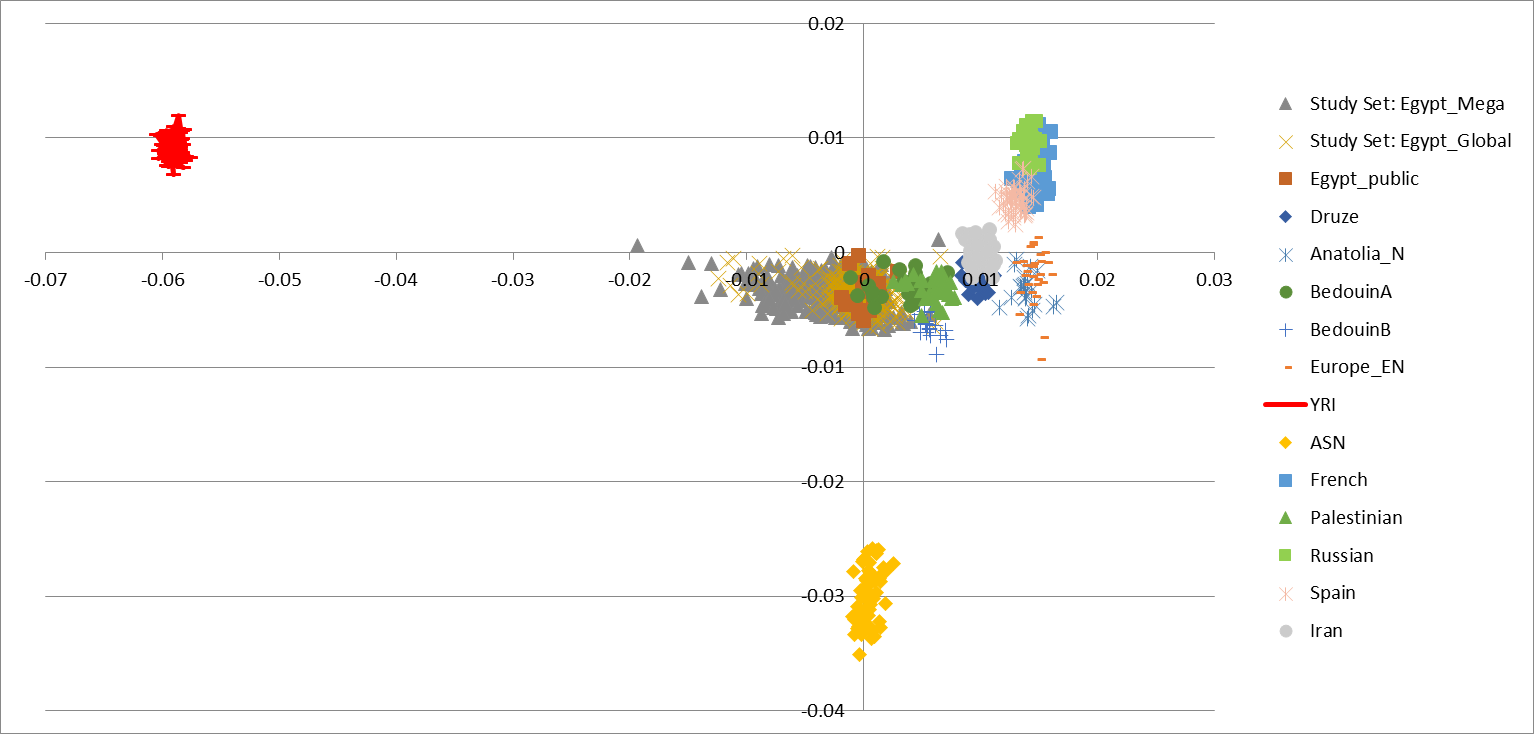


**
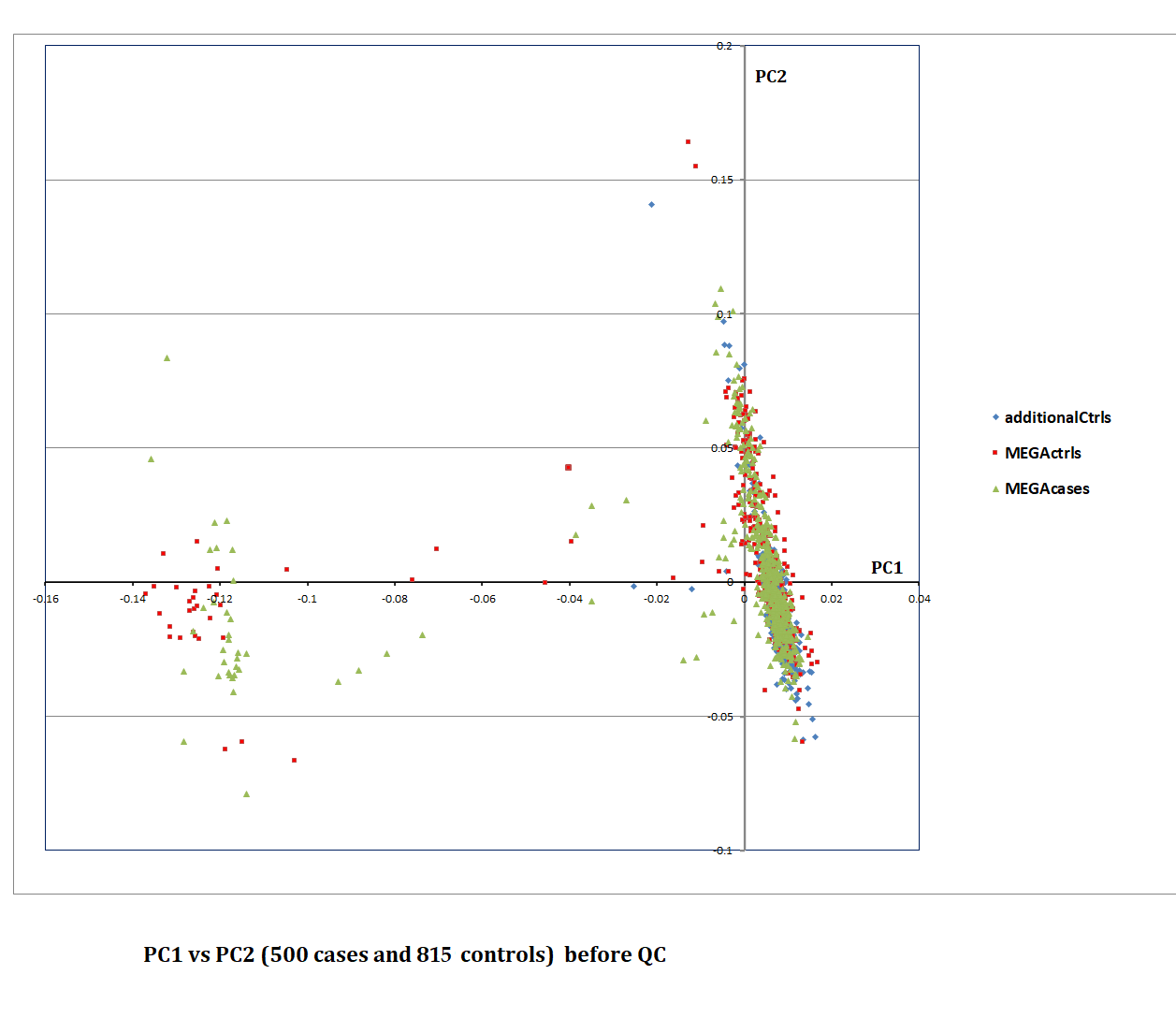
**


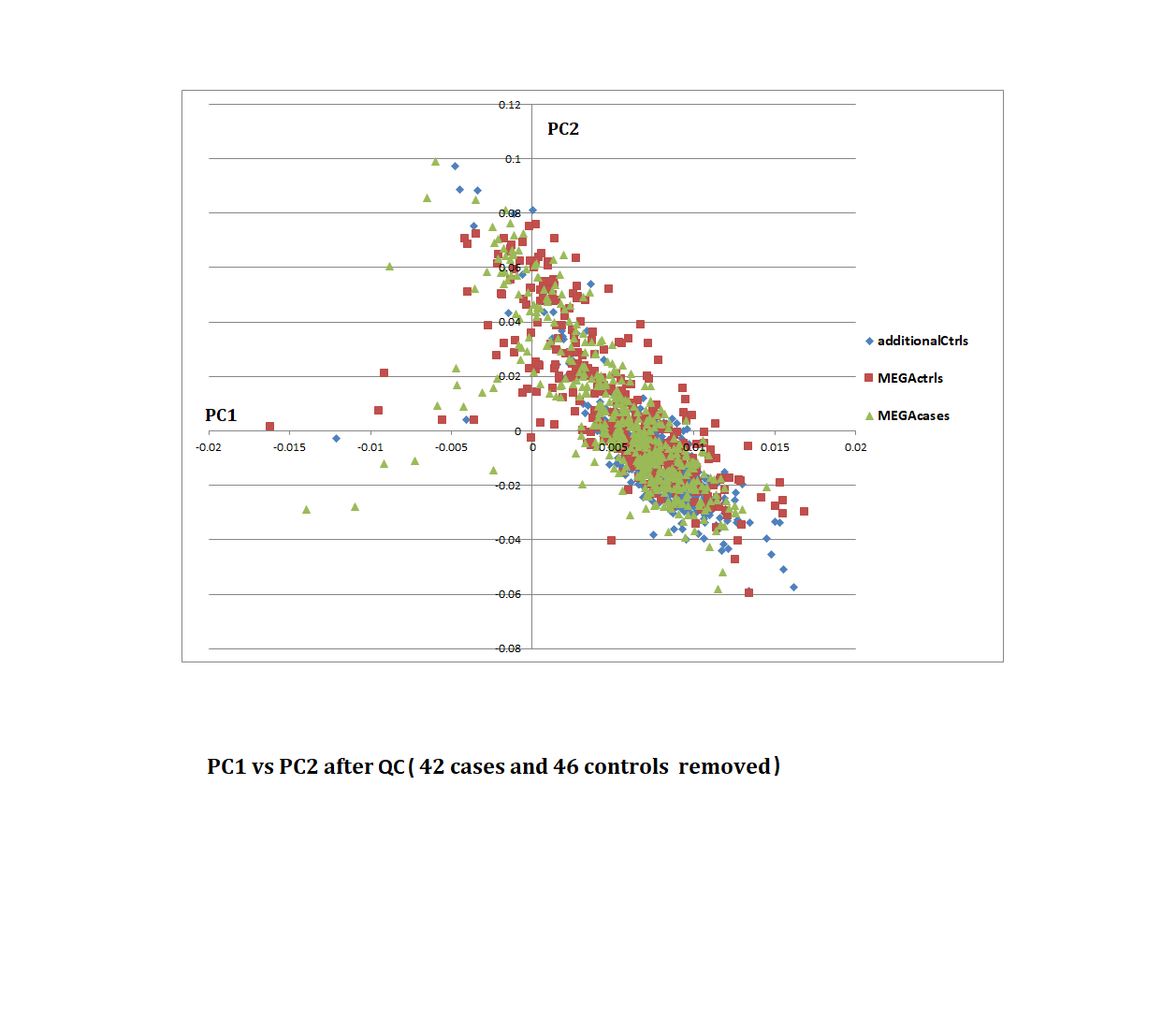


**Supplementary Figure 4.** Admixture cross-validation plot for *K*=1-18 with samples from Near East samples.


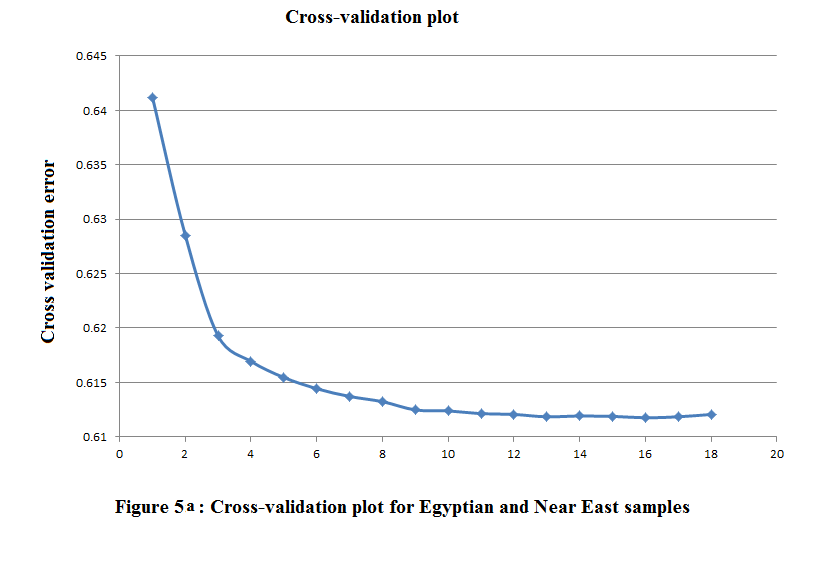


**Number of population (K)**
